# Lymphatic filariasis elimination endgame in an urban Indian setting: the roles of surveillance and residual microfilaremia after mass drug administration

**DOI:** 10.1186/s40249-021-00856-x

**Published:** 2021-05-18

**Authors:** Anjali Modi, Keshav G. Vaishnav, Kailash Kothiya, Neal Alexander

**Affiliations:** 1 Department of Community Medicine, Government Medical College, Surat, Gujarat India; 2 Vector Borne Disease Control (VBDC) Department, Surat Municipal Corporation, Surat, Gujarat India; 3 MRC International Statistics and Epidemiology Group, London School of Hygiene and Tropical Medicine, London, UK

**Keywords:** Lymphatic filariasis, Urban, Migration, Mass drug administration, Surveillance, Epidemiological, Elimination, Residual microfilaremia

## Abstract

**Background:**

To secure the gains of lymphatic filariasis (LF) elimination programs, attention is needed to the ‘residual microfilaremia phase’, in which high-risk populations may be crucial. The present study documents the impact of mass drug administration (MDA) in the urban Indian setting of Surat City, with high rates of in-migration.

**Methods:**

Epidemiological assessment included National Filaria Control Program (NFCP) and World Health Organization recommended routine and pre-MDA microfilaremia surveys respectively. Routine filaria surveys were conducted around the year in approximately 2000–4000 people per month, while pre-MDA surveys were carried out annually among approximately 4000 people from four fixed and four random sites. In 2016, Transmission Assessment Survey (TAS) was done in primary school children. The outcomes were microfilaremia (Mf) and antigen prevalence; more specifically, microfilaremia according to place of birth, in pre-MDA and routine night blood smears (NBS) collected from 2008 to 2015. Prevalence ratios and confidence intervals were calculated.

**Results:**

A total of 25 480 pre-MDA and 306 198 routine NBS were examined during the study. In 2008, the Mf prevalence in the routine survey was 63/18 814 (0.33%), declining to 23/39 717 (0.06%) in 2016. Pre-MDA surveys showed a similar decrease from 47/4184 (1.1%) in 2008 to 12/4042 (0.3%) in 2015. In those born outside Surat, microfilaremia decreased below transmission thresholds, but remained more than treble that of the remainder of the population, in both the pre-MDA surveys [prevalence ratio: 3.17, 95% confidence interval (CI): 1.15–8.72], and the routine surveys (3.31, 95% *CI*: 1.47–7.48). Though the TAS results indicated that MDA endpoints had been reached, sub-group analysis identified that 90% of antigenemic children were from families of high-risk groups.

**Conclusions:**

Extensive long-term epidemiological monitoring suggests that all the urban population, including high-risk groups, have benefitted from the ELF program. To prevent re-establishment of infection in large urban areas with unsanitary conditions conducive to filarial vector breeding, there is need to identify residual microfilaremia by customized surveys in addition to pre-MDA monitoring and TAS. The present findings can be used to develop strategies to prioritize screening, surveillance and plan treatment of high-risk groups after achieving MDA endpoints.

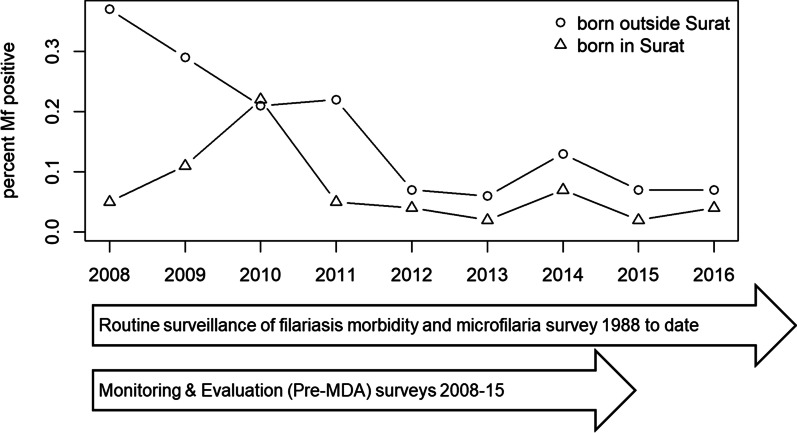

**Supplementary Information:**

The online version contains supplementary material available at 10.1186/s40249-021-00856-x.

## Background

The rapid pace of unplanned urbanization and the lack of proper sanitary conditions in many cities in developing nations enhance the transmission of vector-borne diseases [1–3]. Among these, lymphatic filariasis (LF) is primarily a neglected tropical disease of the poor in disadvantaged peri-urban, urban, and rural areas, causing damage of the lymphatic system leading to long term disfigurement and disability [3].

In May 1997, the World Health Assembly passed a resolution making elimination of lymphatic filariasis (ELF) a public health priority. The global program for ELF (GPELF) depends primarily on mass drug administration (MDA), integrated vector management (IVM), morbidity management and disability prevention (MMDP) [4, 5]. Since 2000, administration of more than 8.2 billion cumulative treatments to approximately 923 million people has helped achieve 43% reduction in the total population endemic for filariasis [6]. Currently, 17 out of the 72 endemic countries with on-going transmission of the mosquito-borne filarial parasites (*Wuchereria bancrofti, Brugia malayi* and *B. timori*) [6, 7] have been validated for elimination of LF, while five countries have stopped MDA and are now under surveillance. India has about 40% of the global filariasis burden and 50% of the global population at risk of infection [8]. *Culex quinquefasciatus* mosquitoes are the principal vectors of LF in India [3, 9].

Considering the huge resources required to provide MDA; programme monitoring, surveillance and evaluation are essential to determine when endpoints have been attained or, conversely, to identify populations and areas requiring renewed efforts [10, 11]. This involves measurement of circulating filarial antigen (CFA) in the human population via a standardized tool known as the Transmission Assessment Survey (TAS). India achieved effective coverage in 90% of the implementing units in 2016, and MDA has been stopped in 94 of 256 endemic districts after passing TAS [7].

The gains achieved to date by ELF programs in India and the world are dependent on preventing the re-establishment of transmission [1, 12, 13]. Global research on urban filariasis is largely of short duration, concentrated in a few small areas [3, 12]; e.g. on India’s eastern coast, with little emphasis on identification of high-risk populations groups [13, 14]. Available review and research articles on ELF and MDA have indicated the need to understand factors that may threaten to reestablish transmission of lymphatic filariasis [1, 3, 14]. Rapid unplanned growth of urban cities and movement of rural population to urban areas in search of jobs, also called internal migration [15], poses one such challenge to coverage of mass drug treatment programs [1, 2]. Another problem is the co-existence of the parasite species *W. bancrofti* with the mosquito vector *Cx. quinquefasciatus* which thrives in unsanitary sewage and drainage conditions, which are common in overcrowded urban areas, home to poor people without secure housing tenancy and who frequently need to move to pursue work [3]. These populations often have an added disadvantage of reduced access to screening and treatment for filariasis unless programs are designed with them in mind [16].

With this background, the present study documents extensive microfilaremia monitoring and surveillance in humans over a decade and shows the impact of MDA on epidemiological indicators in a large urban setting on western coast in the developing country of India, with emphasis on identification of high-risk urban population groups. We envisage programmatic findings from our study will help to guide intervention strategies for maintaining filariasis control in urban areas, especially those which are in the residual microfilaremic phase.

## Methods

### Materials

#### Study settings

**Fig. 1:**
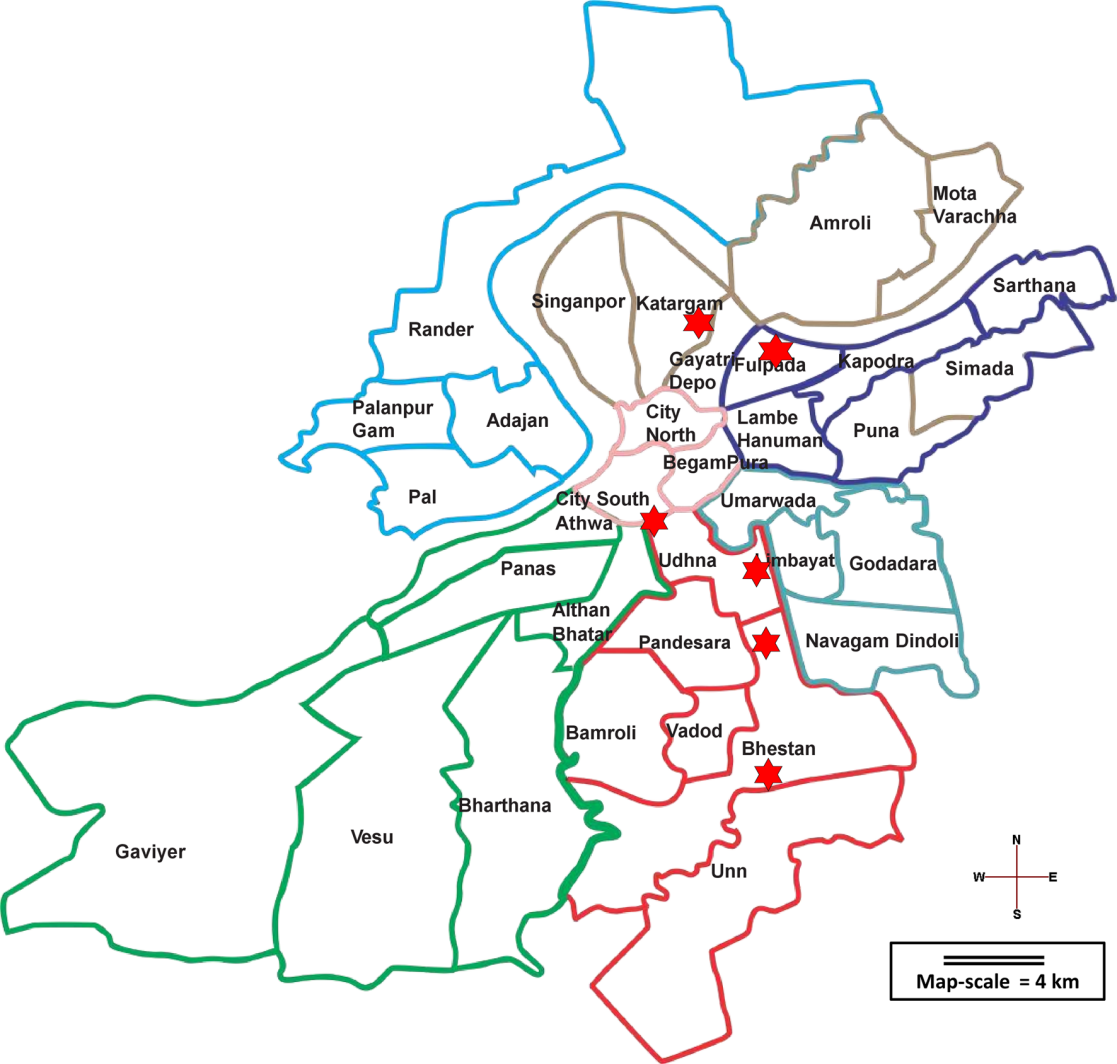
Map of Surat City, Gujarat, India. All LF surveys took place in the seven zones (32 units) shown by different colors on this map of Surat City /Municipal Corporation (SMC). The red stars show the sentinel sites having the highest prevalence of microfilaremia in pre-MDA baseline survey conducted in year 2004 (Table 1). From north to south, the sites are: (1) Utkal Nagar; (2) Ashok Nagar; (3A) Sub-jail; (4A) Tulsi Nagar; (4B) EWS quarters and (3B) Siddharth Nagar. Source: drawn by the authors on the basis of Google Maps depiction of Surat city

The National Filariasis Control Program (NFCP) for India started in the year 1955. On the basis of NFCP’s filariasis endemicity survey, urban Surat (Fig. 1), situated on the western coast of Gujarat state, was allotted one of 47 National Filaria Control Units (FCU) [17], and this unit implemented disease and vector surveillance according to NFCP guidelines and recommendations[8, 18]. The NFCP was merged into the National Vector-Borne Disease Control program (NVBDCP) in 2003–2004. At present, Surat is a metropolitan city with 4.5 million inhabitants in an area of 326 km^2^.

As a signatory to WHA and GPELF, India started MDA in 1996–1997 in the form of a pilot project in Orissa state which was later expanded to country-wide endemic areas with the aim of eliminating filariasis by 2015[9, 11]. Gujarat state including Surat Municipal Corporation (SMC)—the city’s administrative organization—launched MDA with diethylcarbamazine (DEC) and albendazole in 2004 [9, 18, 19]. Surat City was considered as one implementation unit (IU) [4]. The peripheral health workers (PHWs) of the vector-borne disease control department (VBDC) of SMC conducted the ELF program activities under the supervision and guidance of full-time program and health officers. Twelve MDA rounds were carried out between 2004 and 2015. The process (coverage rates) and impact (Mf rate) indicators were monitored and reported every year following NVBDCP and WHO guidelines [4, 18].

A previous independent assessment [20] of MDA in endemic areas of Gujarat state including Surat Corporation found that coverage and compliance were adequate in terms of WHO recommendations [4] and that microfilaremia rate had decreased by 70% in Surat City between 2005 and 2015. Inbuilt program supervision indicates drug distribution above 95% and drug compliance between 70 and 90% in different areas [19].

### Study methods

Monitoring can be considered the component of surveillance whose subject is proactive control measures. Here, the monitoring and evaluation (M&E) or *pre-MDA surveys* were conducted as an annual activity for twelve years (2004–2015) [18]. A total of 4000 NBS were collected from eight sites each year: 500 slides each from four sentinel and four random/spot-check sites (Table 1) [18]. The sentinel sites were identified from the list of city areas with highest microfilaremia and disease prevalence in the baseline survey of year 2004 while spot sites were randomly selected each year (2004–2015) from other city areas irrespective of microfilaremia (Mf) and disease prevalence [4, 20]. In this communication, we present the site-wise trend of Mf rate from year 2008 to 2015 after completion of four initial MDA rounds. This activity is considered adequate to assess the impact of the previous MDA rounds as the GPELF recommends periodic assessment in at least one sentinel site per one million population before (baseline), and then after third and fifth rounds of MDA [4].

**Table 1:** Distribution of microfilaria (Mf)-positives in night blood slides (NBS) collected from sentinel and spot sites during pre-mass drug administration (MDA) surveys

Year	Sentinel sites Mf positive/ slides tested (Mf %)					Spot (random) sites
	Sub jail / Siddharth Nagar (South Zone)	Tulsi Nagar/ EWS quarters, Pandesara (South Zone)	Utkal Nagar (North Zone)	Ashok Nagar (East Zone)	Total	Total
2008	3/512 (0.6)	8/568 (1.4)	13/516 (2.5)	16/509 (3.1)	40/2105 (1.9)	7/2079 (0.3)
2009	3/504 (0.6)	6/530 (1.1)	6/511 (1.2)	37/511 (7.2)	52/2056 (2.5)	9/2069 (0.4)
2010	2/502 (0.4)	5/548 (0.9)	6/519 (1.2)	23/503 (4.6)	36/2072 (1.7)	8/2727 (0.3)
2012	0/500 (0)	1/556 (0.2)	8/516 (1.6)	6/500 (1.2)	15/2072 (0.7)	0/2106 (0)
2014	8/504 (1.6)	2/525 (0.4)	3/516 (0.6)	4/503 (0.8)	17/2048 (0.8)	4/2104 (0.2)
2015	4/501 (0.8)	2/514 (0.4)	0/500 (0)	2/503 (0.4)	8/2018 (0.4)	4/2024 (0.2)

NVBDCP/NFCP recommended preliminary surveys; control by both drug administration and vector management; and follow-up investigations. Adhering to the NFCP guidelines, Surat Filaria Control Unit (FCU) continued the epidemiological assessment of city population by conducting microfilaremia and disease rate surveys every year since 1988 [17]. The present study shows the findings of *NFCP/ routine surveys* conducted between the years 2008–2015. For these surveys, multistage random sampling was employed. Urban Surat was divided first into seven zones, then thirty-two units, followed by hundred wards (Fig. 1). A list of all *mohallas* (small areas) and households was prepared at the ward level. One half of wards were selected at random, subject to no selected ward being selected again in the following three years. From the selected wards, one quarter of *mohallas* were randomly selected. From the list of households in the selected *mohallas* a random selection of 6–10% of the households, with a minimum of 14, was done per *mohalla* following national recommendations for conducting filaria survey [21, 22]. Daytime surveys of selected houses for filaria morbidity cases (lymphadenitis, lymphangitis and elephantoid manifestations) were followed by night blood smear (NBS) collection from all family members of these houses from 9 pm to midnight[19, 20]. Approximately two to four thousand NBS were collected per month (seven teams in seven zones, times five field working days per week, times fifteen households per day).

In 2016, TAS was conducted when WHO and national criteria had been met: (1) five rounds of MDA with effective coverage > 65% had been carried out, and (2) the Mf prevalence of each additional site and pre-MDA spot and sentinel site was less than 1%. The seven zones of Surat City were merged into three evaluation unit (EUs) for effective administration of TAS as guided by WHO and national organizations [4, 18]. The central and east zone were considered as EU1; west, south-west and north zone as EU2; and south and south-east zone as EU3 (Fig. 1). The net primary school enrolment ratio of Surat City was 75.3%—near the recommended threshold of 75%—therefore the school-based surveys strategy was chosen [4, 18]. After selection of TAS strategy, a geographic list of all the city schools was prepared. Then, the WHO-recommended Survey Sample Builder (SSB) software was used for selecting clusters of schools, sample size, sampling fraction and interval from each school, along with pre-determined critical cut-off levels for the number of test positives, to ensure unbiased implementation and evaluation [4, 10, 18]. Only first- and second-year primary-school going children (aged approximately 6–7 years) were considered eligible to be participants for TAS because they have lived most of their lives through MDA and any infection in them indicates recent transmission [4, 10, 18]. A total sample size of approximately seventeen hundred, and a critical cut-off limit of 20 FTS positives, were determined and achieved for each EU (Table 2). For TAS, Filarial Test Strips (FTS; Alere, Scarborough, ME, United States) were procured to detect filarial antigen [23]. The TAS survey was conducted as a four-day long exercise from 3^rd^ to 6^th^ October 2016 after training of PHWs and supporting staff of VBDC department.

**Table 2:** Filarial test strips (FTS) findings among primary school children (6–7 years) of Surat City in transmission assessment survey (TAS) (2016)

Name of the Evaluation Unit	Central & East Zone (EU1)	West, South-West & North Zone (EU2)	South & South-East Zone (EU3)
Estimated number of 6–7 years children in EU	71 969	67 435	72 576
Total number of 6–7 years enrolled in schools	53 982	51 065	54 432
Total number of primary schools in EU	333	340	368
Average number of 6–7 year students per school	162	150	147
Sample size for cluster design	1692	1692	1692
Number of clusters	30	30	30
Sampling fraction	0.41	0.44	0.45
Sampling Interval	2.44	2.26	2.21
Critical cut off	20	20	20
Total FTS done	1850	1865	1849
Total FTS positive	6	10	12
FTS positive born outside Surat	5	10	11
FTS positive in areas with poor sanitation	6 out of 6	10 out of 10	12 out of 12
Total FTS negative	1691	1715	1702
FTS invalid^a^	153	140	135
Antigenemia %	0.35	0.58	0.7

^a^Of these 428 invalid tests, approximately 258 (60%) occurred on the first day (of four) of the TAS survey

All routine (NFCP/NVBDCP) and M&E (pre-MDA) surveys involved NBS collection by peripheral health workers [4]. Briefly, a thick blood smear was prepared from a drop of approximately 20 mm^3^. The smears were Giemsa-stained and examined for microfilariae [4, 20]. Quality assurance was maintained by cross-checking of all positive slides and 5% of negative slides by an independent team of trained staff selected by the State Entomologist. The NFCP staff was trained by SMC Insecticide Officer (IO) for conducting filaria surveys [24].

A line-list of all Mf positive diagnosed in routine or pre-MDA survey was prepared and a detailed case investigation was conducted every year to explore their epidemiological profile. Required investigations, treatments, health counselling and follow-up were done. A predesigned, pre-tested and semi-structured questionnaire was used to collect details including the length of stay at the present area of Surat City, native place of birth and history of visiting native place per year, although only for those Mf-positive. Residential conditions such as open sewage and drainage systems were described for microfilaremic individuals. The routine, pre-MDA and post-MDA surveys were performed irrespective of migration status.

### Study duration, design and participants

**Fig. 2:**
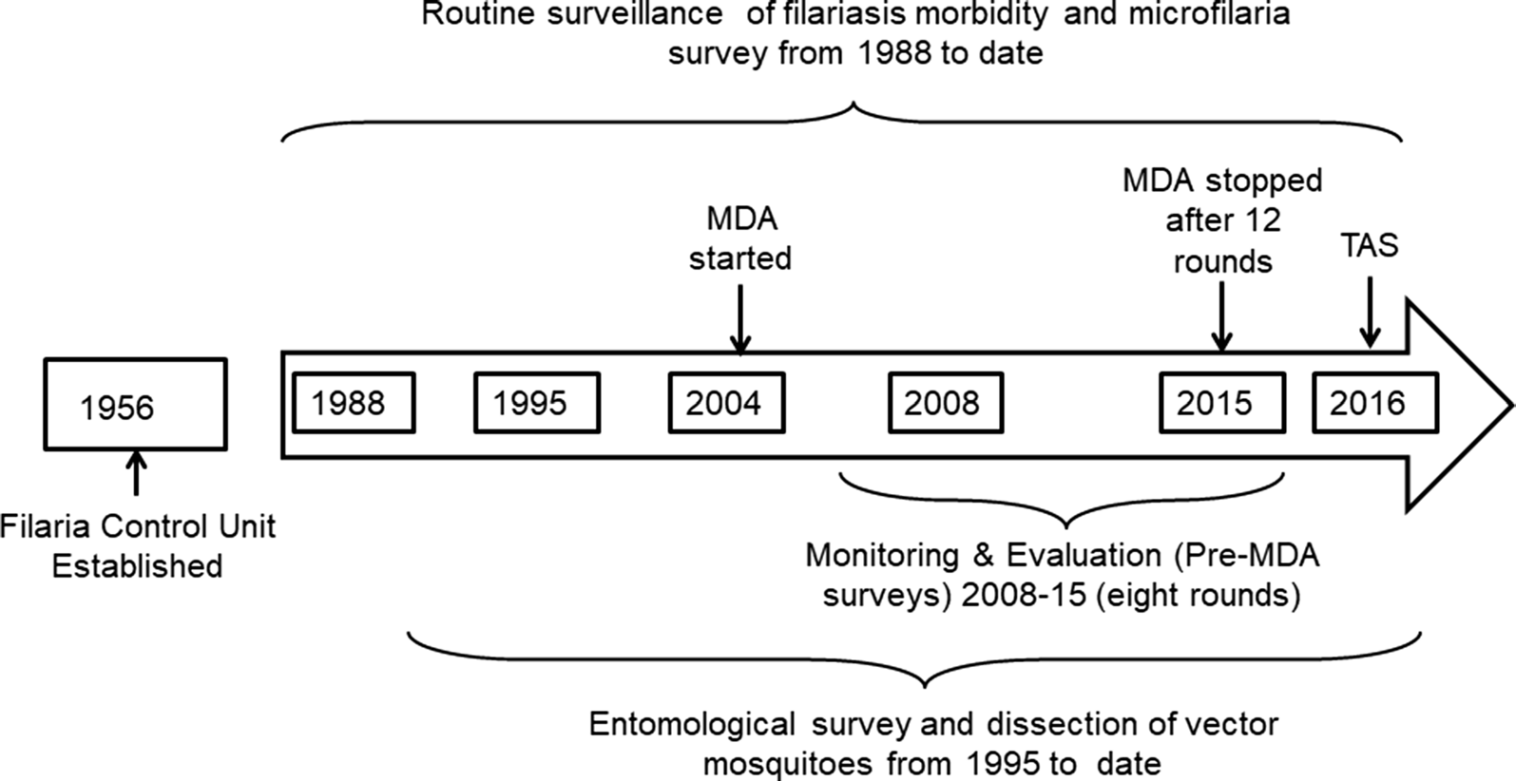
Timeline of the different surveys for elimination of lymphatic filariasis in Surat. WHO collaborative Global Programme to Eliminate Lymphatic Filariasis (GPELF) was adopted in India in 1996 and expanded to involve Surat City in 2004. *MDA* Mass Drug Administration, *TAS* Transmission Assessment Survey

To summarize, epidemiological surveillance of infection and disease in the present study included routine filaria surveys, from 2008 to 2016, M&E (pre-MDA) surveys from 2008 to 2015, and TAS in 2016. City residents except children less than 2 years, pregnant women and severely sick people formed the sampling frame for routine and pre-MDA survey while school-going children aged 6–7 years were selected for TAS. Regular supervision and monitoring was done by program supervisors and managers (Fig. 2). Various terms and indicators used in the study are summarized in the supplementary file for ready reference (Additional file 1).

People from all over Gujarat state (interstate) and other states of the country (intrastate) travel to Surat in search of jobs and business opportunities [15]. In order to meet the objective of identifying subgroups at higher residual risk of microfilaremia, NBS were analyzed according to place of birth captured as village (city), district and state of origin.

*Study variables*: The main outcomes were microfilaremia and antigen prevalence in people over the years 2008 to 2016. The explanatory variable of interest was place of birth i.e. whether or not born in Surat.

### Data management and statistical methods

The PHW collected the data of filaria surveys on pre-designed forms. This was supervised and consolidated to ward-level reports by field supervisors. These results were validated and entered into a Microsoft Excel file. Descriptive analysis was used to report and analyze survey findings; in particular, proportions with confidence intervals. For the latter, to allow for clustering, the intracluster correlation coefficient (ICC) [25] of the proportion Mf-positive was calculated across sentinel sites for each year from 2008 to 2015. These ICCs were then used to estimate design effects to adjust confidence intervals for the prevalence of Mf in humans (50 wards per survey). Prevalence ratios, with confidence intervals and *P* values, were calculated for migration status, aggregated over the period 2008–2015 inclusive, separately for routine and pre-MDA surveys. For this, the method of Katz et al. [26] was used, with the standard error of the logarithm of the prevalence ratio again being inflated via the design effect, taken to be the average of the above year-specific design effects for this period. The R software, version 3.6.3, was used (R Foundation for Statistical Computing, Vienna, Austria).

## Results

### Routine (NFCP) surveys

During the routine surveys, conducted by PHWs between 2008 and 2016 in Surat City, a total of 306 198 NBS (mean 34 021 per year) were examined; of which 353 NBS were Mf-positive (mean 39 per year). This proportion declined from 63/18 814 (0.33%) in 2008 to 23/39 717 (0.06%) in 2016 (Additional file 2). Over the same period, of 306 198 examined, 375 (0.12%) were found to have filarial disease, decreasing from 106/18 814 (0.56%) in 2008 to 20/39 717 (0.05%) in 2016 (Additional file 4).

### Monitoring and evaluation (pre-MDA surveys)

The pre-MDA surveys covered approximately 4000 population from four sentinel and four random/spot sites once a year. Between 2008 and 2015 a total of 25 480 pre-MDA NBS (mean 4247; range: 4042–4799) were examined with 200 Mf-positive. This proportion declined from 47/4184 (1.1%) in 2008 to 12/4042 (0.3%) in 2015 (Table 1 and Additional file 3). Since the beginning of MDA rounds and selection of sites for survey, the sentinel sites had a higher Mf prevalence compared to random sites selected from other city areas. Among the four sentinel sites, Ashok Nagar had the highest Mf prevalence which declined from 3.1% in 2008 to 0.4% in 2015.

### Sub-group analysis of microfilaremia prevalence

**Fig. 3:**
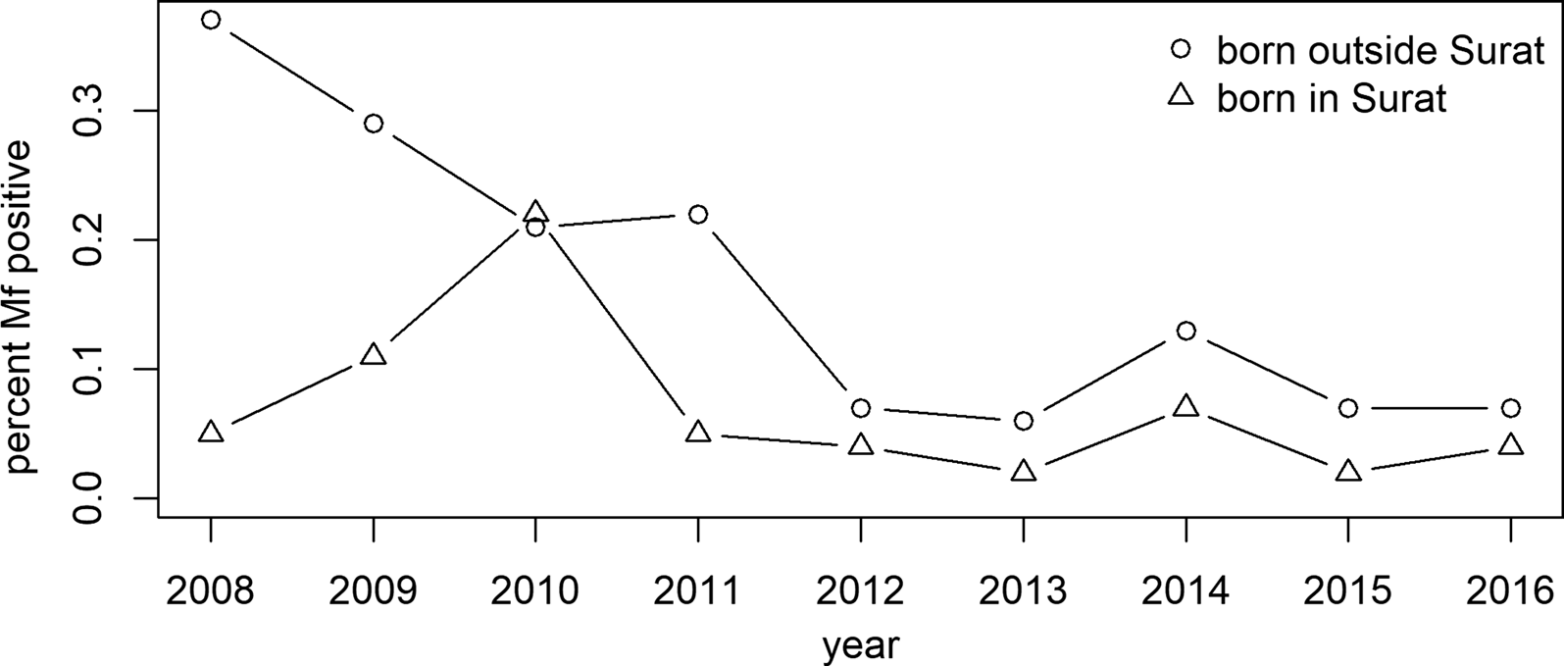
Prevalence of microfilaremia over time according to place of birth: in Surat or elsewhere (migrants). The prevalence of microfilaremia (Mf % or Mf rate) was calculated as the percentage of persons showing microfilaria in their peripheral blood (night blood smears)

**Fig. 4:**
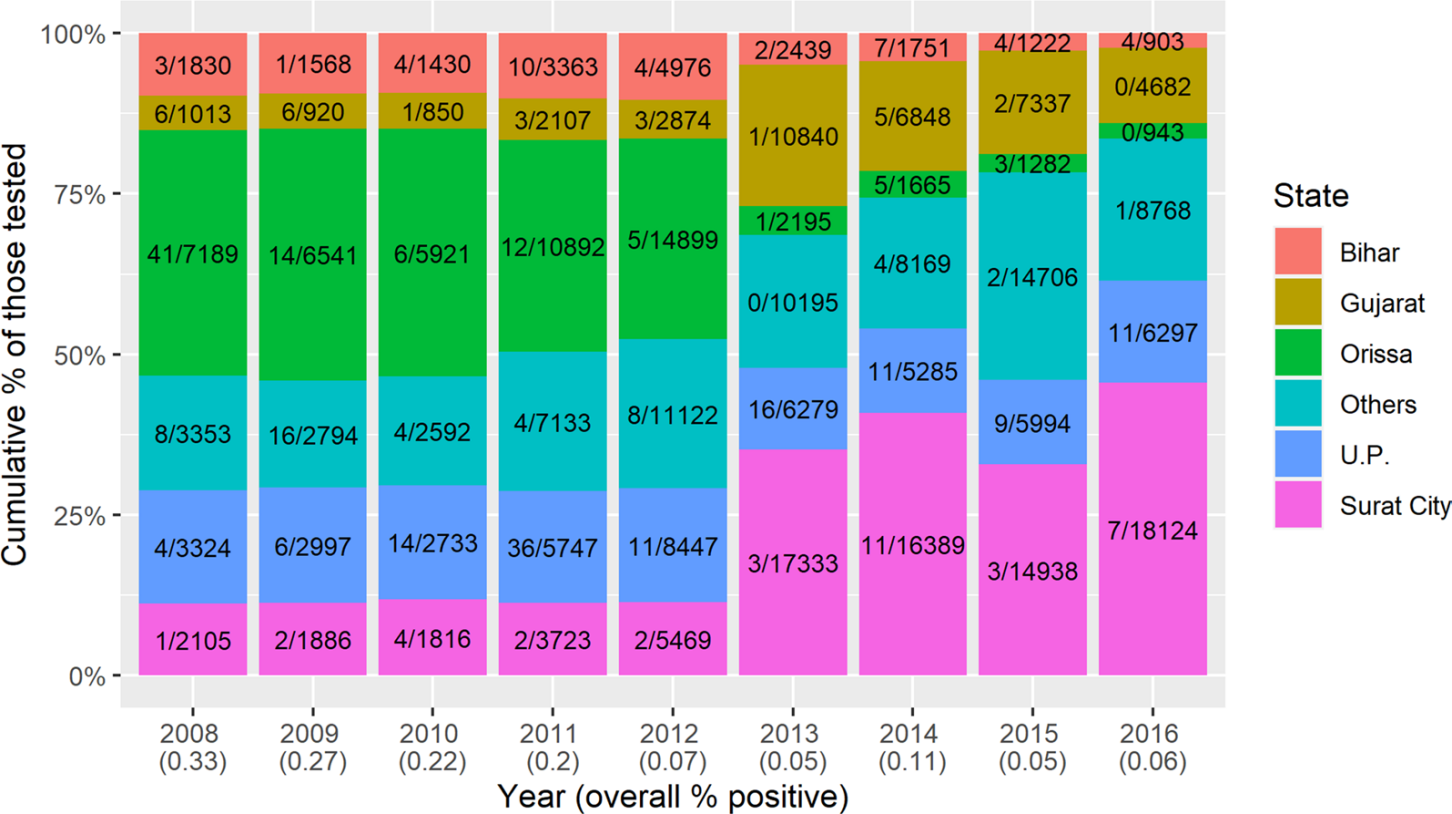
Stacked bar chart of numbers of microfilaremic individuals by state of birth, from the routine surveys in Surat, 2008–16. For example, in 2008, night blood smears from 7,189 individuals born in Orissa were tested for microfilaria, of which 41 were Mf positive

All microfilaremic persons were contacted and line-listed for health advice, home-visits, treatment, adherence, and follow-ups. Out of 306 198 NBS from routine surveys, 224 415 were from those born outside the city (migrants) and 81 783 (24%) from those born in Surat. Of these, 318 (0.14%) and 35 (0.043%), respectively, were microfilaremic (Figs. 3 and 4): a prevalence ratio of 3.31 [95% confidence interval (*CI*) 1.47–7.48, *P* = 0.004]. Although data on living conditions were not systematically collected, positive patients generally lived in highly crowded areas with open drainage systems.

**Fig. 5:**
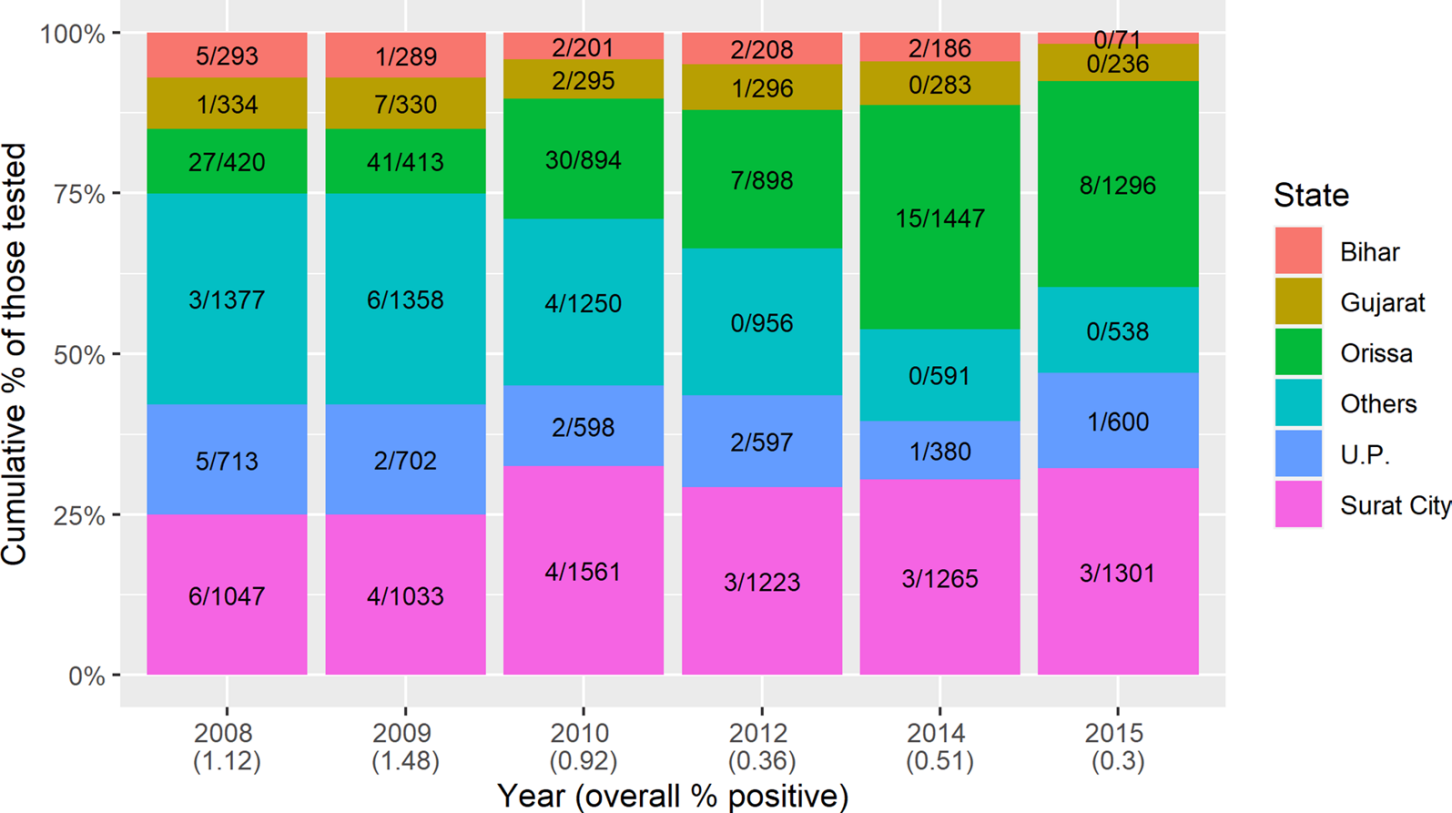
Stacked bar chart of numbers of microfilaremic individuals by state of birth, from the pre-MDA surveys in Surat, 2008–2015. For example, in 2008, night blood smears from 420 individuals born in Orissa were tested for microfilaria, of whom 27 were Mf positive

Corresponding results from the pre-MDA surveys show that out of 25 480 slides examined, 18 050 (71%) were from people born outside Surat and the rest 7430 (29%) were from people born in Surat City (Fig. 5). Among these, 177 (0.98%) and 23 (0.31%) respectively were microfilaremic; a prevalence ratio of 3.17 (95% *CI*: 1.15–8.72; *P* = 0.026).

### TAS or post MDA assessment

In the TAS survey, among school-going children aged 6–7 years, a total of 5564 Filariasis Test Strips (FTS) were performed with six, ten and twelve positive in the three EUs respectively (Table 2). The numbers positive were all less than 20 (the pre-determined cut-off value) in each EU. Of the 28 FTS-positive children, 26 (92%) were not born in Surat (Table 2), and reported returning to visit their place of birth at least once a year. Consistent with the above observations, all microfilaremic children were inhabitants of areas with places conducive to *Culex* vector breeding.

## Discussion

A review of literature shows that studies on ELF and implementation of MDA are very few in large urban areas of India [27] and other endemic parts of the world [1, 14, 28]. The present study describes the MDA program in Surat, a resource-constrained developing country setting, where the challenging task of administering at least 4.5 million treatments (every year for 12 rounds) was undertaken ensuring adequate coverage and compliance [19, 20]. The pre-MDA surveys show that the Mf rate declined by 74% (1.1% in year 2008 and 0.3% in 2015) in Surat City [20]. The routine/NVBDCP surveys indicated a similar decline of 82.7% (0.33% in year 2008 to 0.06% in 2016). These sustained reductions in microfilaremia indicate that MDA has helped to eliminate filariasis from urban areas of Surat and is in accordance with ELF program results in other countries [1, 9].

After successful MDA rounds, an essential challenge to the Global Programme’s success in combating LF is deciding the stopping points for MDA [29]. The WHO recommends TAS surveys to diagnose and determine antigenemia in 6–7 year old children assuming that any infection in these children will detect community transmission in presence of annual mass chemotherapy [4, 10]. In the present study, TAS was conducted with FTS among 1650, 1685 and 1649 (total 5564) children to get an antigenemia prevalence of 0.35, 0.58 and 0.7% in the three EUs respectively (overall 0.54%). Though these results were below the pre-decided target threshold of 2% antigenemia, and established endpoints for MDA in Surat, further sub-group analysis revealed that ninety percent of antigenemic children were from families of populations born outside Surat City and having history of visiting native endemic areas (Table 2). These findings implicate existence of “hidden hot-spots” [11] or residual microfilaremia not detectable by existing recommended GPELF programmatic monitoring.

All positive patients were visited in the field to ensure treatment compliance and it was observed that the living conditions of microfilaremic people, whether born in Surat or not, had poor sanitation and hygiene, e.g. open drains, favoring the *Culex* vector. Simonsen et al. found similar focality in less privileged city populations in his review (2013) of major studies on urban filariasis [3]. Previous findings suggest that even small areas omitted from control programs have the potential to serve as dispersal foci for filariasis including urban areas [13, 14] especially in context of efficient parasite-vector combinations [3].

The MDA achievements in South Gujarat, documented here and elsewhere [12, 19, 20], are tempered by indications that the ‘residual microfilarial infection phase’ or a relatively higher Mf prevalence may exist in certain areas or populations, possibly including those with higher rates of inward migration from endemic areas [14]. On further sub-group analysis of the NBS survey results in the present study, it was observed that annual chemotherapy has succeeded in maintaining decreases in microfilaremia prevalence (Mf rate) in the whole city population, and below 1% in almost all subgroups. However, the prevalence in people born outside Surat or having history of visiting/residing filariasis endemic areas remains treble that of other people (Fig. 3). Populations from relatively high-endemicity northern and eastern states of country were most likely to be microfilaremic (Figs. 4, 5). Some other studies have found similar associations [3, 13, 14, 30] though none has conducted long term comprehensive follow-up of a large city population, both before and after MDA, as reported here. Research in Thailand and the Andaman Islands similarly showed the potential for migration to establish transmission [16, 31]. However, in Sierra Leone and Liberia, filariasis does not seem to have been established by mass migration from rural to urban areas. On the other hand, in these areas the parasite would need to adapt from *Anopheles* to *Culex* vectors [28], a hurdle which does not exist in India where *Cx quinquefasciatus* is the vector of *W. bancrofti*. It is possible that the potential for onward transmission is reduced by their living among people from other parts of the country, with lower prevalence. This reinforces the need for continued vigilance and, in particular to develop and maintain a “comprehensive residual microfilaremic sensitive” urban LF surveillance [1, 12, 15] including entomological surveillance [5] and MDA as appropriate [16]. A systematic review of 42 studies on health and healthcare in India suggests that internal migrants should be considered as vulnerable and specific populations in need of targeted interventions by health systems [15]. We recommend that future research on LF should target on identifying and surveying vulnerable populations in urban areas.

There were several limitations to the present study. Although there was an association between microfilaremia and birth outside Surat, we cannot say with certainty where filarial infections were acquired. In other words, risk of infection within Surat may differ between those born in the city or elsewhere. Another limitation is that health workers are responsible for executing multiple programs during the same field work shifts, which can result in collection of routine NBS at infrequent intervals. Moreover, we have been able to present trend analysis for whole city rather than analysis by site (except sentinel sites of pre-MDA). The surveillance guidance for each program remained constant throughout the period reported here. Overall, this is a strength as the routine/NVBDCP surveys aimed to explore different city populations and areas each year to identify “hot-spots of residual microfilaremia” while pre-MDA surveys focused on sentinel sites to monitor the impact of MDA program. However, for those programs, in particular the routine surveys, which covers one decade, the guidance appropriate at the start of the period may have reduced applicability to the current city, which has changed greatly in terms of culture, demography and sheer size after year 2009 (https://www.suratmunicipal.gov.in/TheCity/City/Stml1).

## Conclusions

These extensive epidemiological studies of urban filariasis suggest that urban populations including vulnerable groups like internal migrants have benefited from programmatic drug treatment and monitoring. It also confirms the global concerns that the overall living conditions and mobility makes people susceptible to higher microfilaremia levels than other populations. We conclude and recommend that after achieving end-points of MDA in large urban areas, ELF programs develop customized “comprehensive residual microfilaremic sensitive” strategies to prioritize existing resources on surveillance and treatment of vulnerable groups living in areas with effective host vector relationship, for these populations’ own benefit and to prevent resurgence in the wider population. The present research can be used as an example to frame strategies to this effect. We also endorse previous findings on the need for guidelines on monitoring migration, with the aim of increasing migrants’ treatment coverage. Standard or WHO recommendations in this regard could further the important goal of maintaining the original gains and preventing re-infection and transmission in high-risk populations; as well as provide an added benefit of enrolment of migrants into the existing health care system.

## Supplementary Information

**Additional file 1.** Table of definitions and terms.

**Additional file 2.** Infection results from routine surveys with place of birth (“state” column), year, number Mf-positive, and number tested in routine surveys.

**Additional file 3.** Pre-MDA results, format as previous file.

**Additional file 4.** Disease results from routine surveys.

## Data Availability

All data generated or analyzed during this study are included in this published article and its supplementary information files.

## Abbreviations

ELF: Elimination of lymphatic filariasis
EU: Evaluation unit
FTS: Filarial test strip
MDA: Mass drug administration
Mf%: Proportion microfilaremic
NBS: Night blood survey
NVBDCP: National Vector-borne Disease Control Program
TAS: Transmission assessment survey
WHO: World Health Organization
