# Supplementary material for: Lymphatic filariasis elimination endgame in an urban Indian setting: the roles of surveillance and residual microfilaremia after mass drug administration
Source: Infect Dis Poverty. 2021 May 18;10:73. doi: 10.1186/s40249-021-00856-x (PMC8130313; doi:10.1186/s40249-021-00856-x)
Supplement: Supplementary file 1 — Additional file 1. Table of definitions and terms. [file 40249_2021_856_MOESM1_ESM.docx]

**Indicator and terms used in the study**

| **Indicator/term** | **Definition** |
| --- | --- |
| Sentinel site | A geographical area, with a population of at least 500 people, selected for collection of parasitological data to monitor the success of the programme. It should remain the same throughout the course of the programme [16]. |
| Spot-check site | A geographical area, with a population of at least 500 people, selected for collection of parasitological data to complement data from sentinel sites. Spot-check sites should be chosen for each assessment and so will change over the course of the programme [16]. |
| Microfilaria prevalence (Mf rate or Mf %) | The microfilaria prevalence (Mf %) was calculated as the percentage of persons showing microfilaria in their peripheral blood (night blood smears) [7, 16, 17]. |
| Disease rate | Proportion of persons manifesting filaria disease [20]. |
| Transmission Assessment Survey (TAS) | A survey designed to measure whether evaluation units have lowered the prevalence of infection to a level where recrudescence is unlikely to occur, even in the absence of MDA interventions [9, 16]. |
| Critical cut-off value | The threshold of infection prevalence below which transmission is likely no longer sustainable, even in the absence of control interventions. The TAS estimates this threshold by the number of antigen-positive cases [9, 16]. |
| Endemic area | Implementation unit where the average resident population, or any subunit of population, has an antigenemia or microfilaraemia prevalence equal to or greater than 2% or 1% respectively [16]. |
| Evaluation unit (EU) | A study area selected for implementation of the transmission assessment survey; can comprise multiple implementation units, or part of an implementation unit [9] less than 2 million [16, 17]. |
| Net-primary school enrolment ratio | The number of children enrolled in primary school that belong to the age group that officially corresponds to primary schooling, divided by the total population of the same age group [16, 17]. |
